# Cross‐Cultural Adaptation of Children's Assessment of Participation and Enjoyment (CAPE) and Preferences for Activities of Children (PAC) in a Danish Context

**DOI:** 10.1111/cch.70377

**Published:** 2026-11-06

**Authors:** Michelle Østergaard, Ida Cecilie Nørgaard, Mette Johansen, Camilla Pedersen, Christina E. Hoei‐Hansen, Søren Hagstrøm, Peter Rosenbaum, Ninna Brix

**Affiliations:** ^1^ Department of Pediatric and Adolescent Medicine Aalborg University Hospital Aalborg Denmark; ^2^ Department of Clinical Medicine Aalborg University Aalborg Denmark; ^3^ Department of Pediatrics and Adolescent Medicine Rigshospitalet Copenhagen Denmark; ^4^ Department of Clinical Medicine University of Copenhagen Denmark; ^5^ CanChild Centre for Childhood‐Onset Disability Research McMaster University Hamilton Ontario Canada

**Keywords:** CAPE/PAC, children with impairments, cross‐cultural adaptation, leisure, measurement, participation, typically developing children

## Abstract

**Background:**

Participation in leisure and everyday activities is central to children's development, health and social inclusion. Although participation is increasingly used as an outcome in clinical practice and research, no cross‐culturally adapted Danish‐language instrument is currently available for systematically assessing children's participation and activity preferences. The Children's Assessment of Participation and Enjoyment and Preferences for Activities of Children (CAPE/PAC) is widely used internationally but requires cross‐cultural adaptation prior to use in Denmark. The aim of this study was to translate and culturally adapt the CAPE/PAC for Danish children and youth with and without impairments.

**Methods:**

Following established cross‐cultural adaptation guidelines, we performed forward translation, synthesis, back translation, harmonization and developer review. To evaluate cultural relevance and comprehensibility, we conducted semi‐structured group interviews with typically developing children (TDC) recruited from a Danish public school, children with impairments (CWI), and their parents, recruited through Happy League (LykkeLiga). Potential new items were mapped to existing CAPE/PAC items, and new items were considered for inclusion if they met a prespecified threshold of ≥ 10%. Linguistic refinements and clarification examples were added where needed.

**Results:**

In total, 126 children participated (109 TDC, 17 CWI). Two original items were replaced due to limited relevance in the Danish context, and contemporary examples were added to nine items to improve clarity and reflect current leisure patterns (e.g., digital participation). Minor linguistic revisions were made to optimize semantic and conceptual equivalence.

**Conclusions:**

We produced a Danish‐translated and cross‐culturally adapted version of the CAPE/PAC to assess participation and activity preferences among children and youth with and without impairments. However, psychometric validation is required prior to widespread clinical and research use.

## Summary

- Participation and preferences are important outcome measures to consider when planning services for children and youth with and without impairments.
- When translating a questionnaire, cultural adaptation to the intended population is essential to ensure conceptual and cultural equivalence.
- The CAPE/PAC has been cross‐culturally adapted for Danish children with and without impairments. However, psychometric evaluation is required before use in clinical practice and research.

## 1 Introduction

Participation, defined in the World Health Organization International Classification of Functioning, Disability and Health (ICF) as ‘involvement in life's situations’, is a central concept in child health and development (World Health Organization 2001). Contemporary participation models distinguish between attendance (the objective ‘being there’) and involvement (the subjective ‘in‐the‐moment experience of participation’), emphasizing that participation involves a dynamic interaction between the child and the environment across everyday contexts (World Health Organization 2001; Imms et al. 2016; King et al. 2004).

Enjoyable leisure participation is associated with physical and mental health, social relationships, skill development and formation of identity and meaning in everyday life (Law 2002; Mahoney et al. 2002; Murphy and Carbone 2008; King et al. 2006; Larson and Verma 1999; Majnemer et al. 2008; Shikako‐Thomas et al. 2008). Ensuring equitable access to meaningful participation is therefore an important clinical and public health goal, particularly for children with impairments (CWI). In this study, CWI refers to a heterogeneous group of children with long‐term physical, intellectual, mental, or sensory impairments who may face environmental, social, or functional barriers (Carbone et al. 2021). Although this population is commonly described in the literature as children with disabilities, we use the term CWI to emphasize our view that impairments should not necessarily result in restrictions in participation. In this context, recent literature emphasizes the importance of assessing both the extent and quality of children's participation and preferences across clinical, research and service settings (Law et al. 2006; Law et al. 2004). However, participation is multifactorial and influenced by individual, environmental and family‐related factors, including age, motor function, cognitive and intellectual abilities, emotional and mental health, available support and societal policies (World Health Organization 2001; King et al. 2006; Shikako‐Thomas et al. 2008; Law et al. 2004; King et al. 2007; King et al. 2003). This complexity underscores the need for systematic approaches that can capture participation comprehensively and guide intervention development (King et al. 2007).

Internationally, several instruments are used to measure children's participation, including the Children's Assessment of Participation and Enjoyment (CAPE), the Preference for Activity (PAC), the Participation and Environment Measure for Children and Youth (PEM‐CY) and the Child and Adolescent Scale of Participation (CASP) (Adair et al. 2018). However, no standardized and validated Danish‐language instrument is currently available. Existing Danish studies predominantly rely on non‐validated self‐reported measures or focus narrowly on physical activity in specific populations, limiting comparability across studies and settings (Fonvig et al. 2021; Melby et al. 2023; Lind et al. 2025; Nordvall‐Lassen et al. 2018; Eilertsen et al. 2024).

The CAPE/PAC is a widely used participation measure aligned with the ICF framework and contemporary participation models and has demonstrated good measurement properties across several languages and contexts (King et al. 2007; Adair et al. 2018; Fink et al. 2016; Longo et al. 2014; Anastasiadi and Tzetzis 2013; Bult et al. 2010; Colón et al. 2008; Ullenhag et al. 2012). For implementation in a Danish context, linguistic translation alone is insufficient to ensure equivalence across cultures. Structured frameworks proposed by Beaton et al. (Beaton et al. 1976) and the International Society for Pharmacoeconomics and Outcomes Research (ISPOR) Task Force (Wild et al. 2005) provide and emphasize systematic procedures to ensure semantic, idiomatic, experiential and conceptual equivalence across contexts (Colón et al. 2008; Ullenhag et al. 2012; Beaton et al. 1976; Wild et al. 2005).

Therefore, the aim of this study was to translate and cross‐culturally adapt the CAPE/PAC for use among Danish children and youth with and without impairments, as a foundation for subsequent psychometric evaluation.

## 2 Materials and Methods

### 2.1 The Instrument

The CAPE/PAC was developed in Canada in 2004 to assess participation and recreational activities outside school among children and youth aged 6–21 years, with and without impairments. The instruments comprise 55 activities categorized into five activity types: recreational, active physical, social, skill‐based, and self‐improvement/educational. Activities are further classified as either formal or informal. The CAPE assesses participation across five dimensions: diversity, frequency, with whom, where, and enjoyment (see Table 1). The PAC addresses preferences using three response options: (1) I would not like to do at all, (2) I would sort of like to do, and (3) I would really like to do (King et al. 2004).

**TABLE 1:** Overview of CAPE scores within each dimension.

CAPE dimensions	Raw data	Score	Range
Diversity	Yes or no if activity was done	The sum of performed activities	0 (low overall diversity) to 55 (high overall diversity)
Intensity	1 = once/4 months 2 = twice/4 months 3 = once/month 4 = 2–3 times/month 5 = once/week 6 = 2–3 times/week 7 = once/day	The sum of “intensity” scores divided by the total number of items in the desired scale.	1 (low overall intensity) to 7 (high overall intensity)
With whom	1 = alone 2 = close family 3 = relatives 4 = friends 5 = others	The sum of “with whom” scores divided by the diversity score in the desired scale.	1 (solitary) to 5 (social)
Where	1 = at home 2 = relative homes 3 = neighbourhood 4 = school 5 = community 6 = beyond your community	The sum of “where” scores divided by the diversity score in the desired scale.	1 (home‐based) to 6 (community‐based)
Enjoyment	1 = not at all 2 = somewhat 3 = pretty much 4 = very much 5 = love it	The sum of “enjoyment” scores divided by the diversity score in the desired scale.	1 (low overall enjoyment) to 5 (high overall enjoyment)

*Note:* The desired scoring scales include overall, domain‐specific, or activity‐type scores.

Abbreviation: CAPE, Children's Assessment of Participation and Enjoyment.

Both instruments can be completed independently or together, either self‐administered or by interview. Administration time is approximately 30–45 min for the CAPE and 15–20 min for the PAC. However, when used together, the CAPE/PAC provides a more comprehensive understanding of a child's participation profile (King et al. 2004).

Scores can be calculated at three levels: overall, domain, and activity type. The overall score represents the total participation across all items within the five dimensions. Domain scores distinguish between formal (15 items) and informal (40 items) activities, whereas activity type scores distinguish between recreational (12 items), active physical (13 items), social (10 items), skill‐based (10 items), and self‐improvement (10 items) activities (King et al. 2004). For each activity type, diversity scores are calculated as the number of items performed. Intensity scores are calculated as the sum of intensity scores divided by the total number of items within the respective activity type. With whom, where, and enjoyment scores are calculated as the sum of responses divided by the diversity score (Table 1). The authors developed a Danish electronic scoring system in Excel to handle all possible scores.

### 2.2 Study Design and Participants

This cross‐sectional study included two groups of participants: typically developing children (TDC) recruited from a Danish public school and CWI recruited through the Happy League (LykkeLiga), a national inclusive sports community adapted for CWI (LykkeLiga 2025). Inclusion criteria for TDC were age 6–16 years, enrolment in a Danish public school, and Danish language proficiency. For CWI, the criteria were age 6–21 years, participation in the Happy League programme, and Danish language proficiency. Parents of CWI were invited to contribute to the interview‐based cultural adaptation component. The CWI were assigned to grade levels based on their age (e.g., 8 year olds to second grade and 10 year olds to fourth grade).

### 2.3 Ethics

The study was registered and approved by the regional authority responsible for data protection (approval ID: K2024‐181) and conducted in accordance with the Helsinki Declaration. The school management granted written permission to conduct semi‐structured interviews and administer the CAPE diversity questionnaire. Teachers provided parents with visit and study information. Children provided verbal consent to participate in the interviews. Interview participation was voluntary for TDC and CWI, and participants could withdraw without consequences. Because the questionnaire was completed anonymously and no identifiable personal data were collected, applicable Danish regulations did not require written parental consent. However, regardless of the child's age, parents of CWI provided written informed consent to participate in the CAPE questionnaire as part of a pilot study event. Children aged 15 years or older could additionally choose to provide their own written consent.

### 2.4 Procedure

#### 2.4.1 Translation

First, written permission to translate the CAPE/PAC into Danish was obtained from CanChild, McMaster University, Hamilton, Canada. A formalized translation procedure, recommended by Beaton et al. (Beaton et al. 1976), was applied in accordance with the International Society for Pharmacoeconomics and Outcomes Research (ISPOR) guidelines for the translation and cultural adaptation of patient‐reported outcomes (Beaton et al. 1976; Wild et al. 2005). Three forward translators independently translated the English version into Danish. A consensus version was then synthesized at a review meeting addressing discrepancies. This version was back‐translated into English by two professional bilingual translators (native English speakers) who were blinded to the original instruments and study objectives. Both back translators had lived experience as parents of CWI, which contributed to a stakeholder‐informed perspective during the translation process (Beresford and Russo 2020). The back‐translated versions were compared with the original version to ensure semantic and conceptual equivalence. Discrepancies were discussed and resolved by a harmonization committee comprising the advisory board and back translators. The final back translation was reviewed by CanChild, and a written report documenting the translation process was approved by all members in accordance with the guidelines of Beaton et al. and ISPOR (Beaton et al. 1976; Wild et al. 2005).

#### 2.4.2 Cultural Adaptation

To assess the cultural relevance of the CAPE/PAC activities in a Danish context, four semi‐structured group interviews were conducted across four public school classes representing Grades 2, 4, 6, and 9. Each interview involved approximately 25 students and lasted 45 to 60 min.

Students were asked about the activities in which they usually participate, both outdoors and indoors, during spring/summer and autumn/winter. One researcher facilitated the interviews, while another documented all activities mentioned. A similar semi‐structured group interview was conducted with seven CWI and eight parents of CWI participating in the Happy League during a training session. All interviews were conducted in September 2024, and data were based solely on field notes collected during the interviews. Two reviewers independently compared the activities identified by TDC, CWI, and parents of CWI with the original CAPE/PAC items, using the Item Descriptions and Distribution from the CAPE/PAC manual to determine whether each activity represented a distinct participation activity or an example of an existing item. Discrepancies were resolved by consensus. Activities not represented in the original instrument were listed as proposed new items.

In November 2024, TDC and CWI completed the CAPE diversity dimension supplemented with a list of proposed new items. The PAC preference component was not evaluated separately, as CAPE and PAC share the same set of activities. Therefore, adaptations identified through the evaluation of CAPE diversity were applied consistently to both instruments. TDC were recruited from the same public school, with one class from each Grades 2, 4, 6, 8, and 9, whereas CWI were recruited through the Happy League. Although the semi‐structured interviews and questionnaire samples were drawn from the same school and spanned an overlapping range of grade levels, they did not consist of identical classes. TDC completed the questionnaire independently during school hours under supervision from the research team, who provided clarification as needed. CWI completed it during an event with parental support available if needed, as well as support from the research team. The event was organized by the Happy League in collaboration with the Research Unit for Paediatric and Adolescent Medicine at Aalborg University Hospital.

### 2.5 Data Analysis

Descriptive analyses were performed to calculate the total counts and percentages of activities reported by TDC, CWI, and the combined cohort. For original activities, a prespecified ≥ 10% threshold in the total cohort was used as a pragmatic criterion to identify activities potentially relevant to culture, consistent with prior Scandinavian adaptation work (Ullenhag et al. 2012). The threshold supported decision‐making during adaptation. However, exceptions could be made by consensus if activities were highly prevalent within a subgroup. For proposed new activities, the threshold was used as a guide, with decisions primarily based on their relation to existing activities and definitions in the CAPE/PAC Manual. Data analyses were conducted using STATA/SE version 17.0.

### 2.6 Missing Data

Participants with more than 20% missing data on either the CAPE or the additional questionnaire were excluded from the percentage analyses in accordance with the CAPE/PAC manual (King et al. 2004). Datasets with less than 20% missing responses were retained for analysis.

## 3 Results

### 3.1 Participants

A total of 126 children completed the CAPE diversity dimension along with a supplementary list of proposed new items. Their characteristics are summarized in Table 2. Due to missing data exceeding 20%, two participants were excluded from the CAPE diversity analysis, and three from the supplementary questionnaire analysis. Consequently, 124 children (107 TDC and 17 CWI) were included in the CAPE diversity analysis, and 123 children (107 TDC and 16 CWI) were included in the supplementary questionnaire analysis.

**TABLE 2:** Participants' characteristics.

	TDC	CWI
	*n = 109*	*n = 17*
Sex*, n (%)*		
Boy	59 (54)	11 (65)
Education*, n (%)*		
Public school	109 (100)	1 (6)
Public school, special class	—	7 (41)
Special school	—	8 (47)
Special boarding school	—	1 (6)
Grade*, n (%)*		
Second	22 (20)	1 (6)
Fourth	27 (25)	3 (18)
Fifth	—	2 (12)
Sixth	24 (22)	3 (18)
Seventh	—	3 (18)
Eighth	18 (17)	2 (12)
Ninth	18 (17)	2 (12)
Impairment type, *n (%)*		
Autism spectrum disorder	—	6 (35)
Intellectual impairment/developmental delay	—	7 (41)
Genetic disorder	—	3 (18)
Downs syndrome	—	3 (18)
Neuromuscular disorder	—	2 (12)
ADHD	—	3 (18)
Number of impairments, *n (%)*		
1	—	6 (35)
2	—	7 (41)
≥ 3	—	4 (24)
GMFCS, *n (%)*		
Level 1	—	12 (71)
Level 2	—	4 (24)
Level 3	—	1 (6)

Abbreviations: ADHD, attention deficit hyperactivity disorder; CWI, children with impairments; GMFCS, gross motor function classification System; TDC, typically developing children.

### 3.2 Cultural Differences

The distribution of children's responses to the items in the original CAPE is presented in Supporting Information A. Using the predetermined 10% inclusion threshold, three CAPE items did not meet the criterion and were considered for removal. These were item 19 (“Riding (a horse)”), item 23 (“Taking art lessons”), and item 27 (“Taking music lessons”). However, item 19 was retained as it was reported by 29% of CWI.

Table 3 summarizes proposed new items derived from the semi‐structured interviews, all of which exceeded the ≥ 10% inclusion threshold. To maintain a total of 55 items and to ensure international comparison, two new items were included in the Danish version. Specifically, item 23 (“Taking art lessons”) was replaced with “Doing creative activities online (e.g., programming and making music)”, and item 27 (“Taking music lessons”) was replaced with “Going to a café/restaurant”. The two new items were classified as a skill‐based activity and a social activity, respectively. Item 23 was classified within the informal domain, whereas Item 27 remained within the formal domain. Illustrations matching the original visual style were developed for the newly added items.

**TABLE 3:** Proposed new items for the Danish version from semi‐structured interviews.

Item	Activity, *n (%)*	Engaging in activities, *n (%)*			Proposed as examples in the original items
		TDC *n = 107*	CWI *n = 16*	In total *n = 123*	
1	Doing creative activities online (e.g., programming and making music)	25 (23)	3 (19)	**28 (23)** ^a^	
2	Having a sleepover	79 (74)	6 (38)	85 (69)	Item 9: visiting
3	Talking with family at home	104 (97)	16 (100)	120 (98)	
4	Running (alone)	67 (63)	5 (31)	72 (59)	Item 40: doing individual physical activities
5	Riding a motocross bike	14 (13)	5 (31)	19 (15)	
6	Riding an electric scooter	45 (42)	2 (13)	47 (38)	
7	Riding a scooter	17 (16)	2 (13)	19 (15)	
8	Bathing or swimming in a swimming hall, the sea, a swimming pool, or a hot tub.	84 (79)	16 (100)	100 (81)	Item 34: doing water sports
9	Using telephone or iPad (e.g., YouTube, playing games, Facebook, Instagram, TikTok)	105 (98)	16 (100)	121 (98)	Item 5: playing on computer
10	Going to the beach	60 (56)	16 (100)	76 (62)	Item 46: going on a full‐day outing
11	Going to a picnic	19 (18)	8 (50)	27 (22)	Item 46: going on a full‐day outing
12	Attending a handball, football or ice hockey match	68 (64)	11 (69)	79 (64)	Item 45: going to a live event
13	Going on a camp trip/colony	43 (40)	2 (13)	45 (37)	
14	Going to a café/restaurant	96 (90)	16 (100)	**112 (91)** ^a^	
15	Going to the forest	80 (75)	14 (88)	94 (76)	Item 46: going on a full‐day outing
16	Enjoying sweets/waffles/ice cream or something else	105 (98)	16 (100)	121 (98)	
17	Taking care of a sibling	56 (52)	1 (6)	57 (46)	Item 50: doing chores
18	Cooking	86 (80)	10 (63)	96 (78)	Item 52: making food
19	Playing rounders	28 (26)	7 (44)	35 (29)	Item 37: playing games
20	Playing tag	62 (58)	12 (75)	74 (60)	Item 37: playing games
21	Enjoying warm cocoa	86 (80)	10 (63)	96 (78)	
22	Going on summer vacation	66 (62)	16 (100)	82 (67)	
23	Going on a ski holiday	13 (12)	1 (6)	14 (11)	
24	Staying at a cottage	63 (59)	12 (75)	75 (61)	

Abbreviations: CWI, children with impairments; TDC, typically developing children.

^a^ Indicates activities included as new items in the proposed Danish version.

Additionally, 12 of the proposed new items were conceptually consistent with existing CAPE items and were therefore incorporated as additional examples (e.g., “Running alone” added under “Doing individual physical activities”). In accordance with the Item Descriptions and Distribution in the CAPE/PAC manual, 10 potential items were not retained as separate items. Three did not meet the manual's conceptual criteria for a distinct participation activity (Items 5–7) and six did not describe a specific participation activity (Items 1, 16, 21–24). One activity (Item 13) was not retained because it is organized within a school context in Denmark.

### 3.3 Adaptation to Changing Times

To reflect technological and lifestyle changes since the development of CAPE in 2004, several items were updated to include contemporary examples. For instance, Item 5 (“Playing computer or video games”) was expanded to “Playing computer, phone, tablet, PlayStation, X‐box”. Item 6 (“Talking on the phone”) was revised to “Talking on the phone (e.g., calls, FaceTime, etc.),” and Item 44 (“Watching TV or a rented movie”) was updated to “Watching TV or a movie.”

### 3.4 Linguistic Changes

To enhance clarity and linguistic accuracy, 11 items were rephrased or supplemented with written examples, while retaining their original conceptual meaning. This applied to Items 3, 11, 14, 15, 20, 25, 33, 35, 36, 43, and 51. For example, item 25 (“Getting extra help for schoolwork from a tutor”) was translated as “Få ekstra hjælp til lektier fra en tutor/vejleder”, since we do not expect all children to know what a tutor is. “Vejleder” is a more common Danish term for a person who guides in an educational setting. A comprehensive written report documenting all translation decisions, cultural considerations, and linguistic adaptations is provided in the Supporting Information B.

Overall, the results support the cultural relevance and linguistic appropriateness of the proposed Danish version of the CAPE/PAC.

### 3.5 The Scoring Implications of Item Replacement

Reclassifying Item 27, “Going to a café/restaurant,” from the skill‐based to the social activity type changed the intensity‐score calculation for both activity types. The social activity type increased from 10 to 11 items, whereas the skill‐based activity type decreased from 10 to nine items. Accordingly, the denominators used to calculate the CAPE intensity and PAC preference scores were adjusted to 11 for social activities and nine for skill‐based activities. Furthermore, reclassifying Item 23 from the formal to the informal domain changed the number of items used to calculate domain intensity scores to 14 and 41, respectively.

## 4 Discussion

Measuring participation among children and youth is essential in both clinical practice and research (Law et al. 2006; Law et al. 2004). This study translated and cross‐culturally adapted the CAPE/PAC for use among Danish children and youth with and without impairments, addressing linguistic, cultural, and contextual factors relevant to contemporary participation. Based on semi‐structured interviews and consensus discussions, two items were replaced due to limited relevance in the Danish context, and several items were supplemented with contemporary examples to reflect current leisure patterns.

Participation is influenced not only by physical abilities and environmental opportunities but also by cognitive, intellectual, and mental health factors. Children's executive functioning, communication skills, emotional regulation, and psychosocial well‐being may substantially shape both attendance and involvement in activities (King et al. 2003). Instruments assessing participation should therefore be applicable across a wide range of cognitive and psychological functioning to ensure inclusivity and accurate representation of children's everyday lives.

Internationally, CAPE/PAC is the most widely used instrument for this purpose, providing valuable insights from children and youth (Adair et al. 2018; Imms 2008). Despite its widespread use, this study is the first to provide a cross‐culturally adapted Danish version of the CAPE/PAC for children and youth. When preparing a questionnaire for use in another setting, cross‐cultural adaptation involves addressing both linguistic and cultural considerations to ensure that measures of constructs influenced by culture and environment accurately reflect the intended population's context (Beaton et al. 1976). Particularly, this is important for participation measures, which are influenced by culture and environment.

### 4.1 The Importance of Cross‐Cultural Adaptation

Cultural differences across populations may lead the same questionnaire to measure different constructs if it is not cross‐culturally adapted (Beaton et al. 1976). For example, an activity in one culture may be unknown or rare in another, and children may overreport socially desirable participation while underreporting non‐normative activities. In this context, Beaton et al. (Beaton et al. 1976) emphasize that a direct translation ensures only semantic equivalence; however, the meaning of items may be lost if idiomatic, conceptual, and experiential equivalence are not achieved (Beaton et al. 1976). Therefore, cultural adaptation is crucial to ensure that the items are appropriate for the intended population and that the instrument assesses children's actual participation rather than risking cultural misunderstandings. Likewise, Beaton et al. (Beaton et al. 1976) address that a cultural adaptation will increase validity and reliability while ensuring that the results remain suitable for international comparisons (Beaton et al. 1976). Finally, psychometric testing should be conducted on the adapted questionnaire to ensure its validity (Epstein et al. 2015).

### 4.2 Cultural Relevance and Adaptation Decisions

A small number of original CAPE/PAC activities fell below the predefined threshold in the combined cohort. Item retention decisions were informed by subgroup relevance (e.g., retaining “Riding (on a horse)” due to higher endorsement among CWI) in line with the instrument's intended applicability across children with and without impairments. Although multiple proposed new activities exceeded the threshold, several were not aligned with the instrument's conceptual focus. The CAPE/PAC manual's activity descriptions and guidelines were used to evaluate whether activities identified during interviews correspond to existing items and meet the manual's criteria for participation activities. As frequency of activities alone does not establish a distinct participation construct, some commonly reported activities were not retained as separate items. This limited unnecessary item proliferation while preserving the instrument's conceptual structure and international comparability.

The reclassification of Items 23 and 27 was intended to improve their conceptual alignment with the most appropriate activity type. However, the changes in item composition and scoring may limit direct comparability with studies using the original CAPE/PAC or versions with different item structures.

The inclusion of “Doing creative activities online (e.g., programming and making music)” reflects the increasing role of digital and technology‐based participation in Danish children's everyday lives. Although reported by a minority of participants, the item was considered conceptually relevant given the integration of digital activities across leisure contexts.

### 4.3 Linguistic Changes and Adaptation to Changing Times

Children's leisure patterns—particularly digital and screen‐based participation—have changed substantially since the development of the instrument. Rather than altering constructs, we updated item examples (e.g., Item 5 now includes phone, tablet, PlayStation, and Xbox, rather than the term ‘video game’) to improve face validity and comprehension among contemporary Danish children, while retaining conceptual equivalence with the original instrument.

### 4.4 Compared to Other Countries

The results of the present study demonstrate context‐specific distinctions between details of the content of the Canadian and Danish CAPE/PAC, reflecting differences in contemporary participation, linguistic interpretation, and underlying cultural norms. Consistent with adaptations in other Scandinavian countries, the Danish version required minor cultural and contextual modifications. These findings support the instrument's cross‐cultural applicability in similar sociocultural contexts. Low participation in Item 23 (“Taking art lessons”) has similarly been reported, leading to its exclusion in the Swedish version but retention in the Norwegian version (Ullenhag et al. 2012; Nordtorp et al. 2013; Hoberg and Nyquist 2008). In both the Swedish and Norwegian versions, contextual examples were added to enhance relevance and comprehension, suggesting a high degree of cross‐cultural applicability of CAPE/PAC within Scandinavian contexts. However, a systematic review by Abid et al. (Abid et al. 2023) reveals that the majority of adapted and translated versions of the CAPE did not meet all criteria for good quality according to translation and cross‐cultural guidelines (Abid et al. 2023).

### 4.5 Strengths and Limitations

A key strength is that cultural adaptation was informed directly by Danish children and families, the intended end users of the instrument. The study also followed established translation procedures and included developer review. Several limitations must be considered. First, the small CWI sample recruited from an inclusive sports programme may have limited generalisability, as the impairments may not represent the broader CWI population and this group may be more active than other CWIs. In addition, communication ability, cognitive functioning, and support needs were not collected. We acknowledge that these characteristics may influence both participation patterns and the comprehensibility of the instrument. Second, TDC were recruited from a single public school in a suburban area. Consequently, the sample does not account for potential geographical, socioeconomic, and school‐type differences that may influence the range of leisure activities relevant to Danish children. This may have limited the generalisability of the identified activities to the broader TDC population. Additionally, the study did not systematically assess cognitive and mental health characteristics among TDC, which may influence participation patterns and should be addressed in future validation work. Another limitation is that the PAC preference was not independently evaluated. Although CAPE and PAC share the same set of activities, further psychometric and feasibility testing of the adapted Danish CAPE/PAC instrument is required. Finally, the presence of English words in some illustrations may influence interpretation and should be considered in future iterations.

The present study provides a methodological foundation for evaluating the Danish CAPE/PAC's measurement properties. Next steps include evaluation of feasibility in clinical settings, test–retest reliability, and construct validity, including expected differences by impairment status and age group.

## 5 Conclusion

This study provides a Danish cross‐culturally adapted version of the CAPE/PAC and establishes a foundation for subsequent psychometric evaluation before clinical or research use to assess participation in leisure activities among Danish children with and without impairments.

## Author Contributions

**Michelle Østergaard:** writing – original draft, data curation, investigation, project administration, visualization, methodology, formal analysis, resources. **Ida Cecilie Nørgaard:** writing – review and editing, data curation. **Mette Johansen:** conceptualization, writing – review and editing, supervision, methodology, data curation. **Camilla Pedersen:** writing – review and editing, investigation, data curation. **Christina E. Hoei‐Hansen:** writing – review and editing, supervision, methodology, conceptualization. **Søren Hagstrøm:** supervision, writing – review and editing, methodology, conceptualization. **Peter Rosenbaum:** supervision, writing – review and editing, conceptualization. **Ninna Brix:** conceptualization, methodology, writing – review and editing, supervision, data curation.

## Funding

The authors have nothing to report.

## Ethics Statement

The study was approved by the local ethics committee (K2024‐181) and conducted in accordance with the Helsinki Declaration.

## Consent

Written parental consent was waived in accordance with Danish data protection regulations, as only anonymized survey data were collected.

## Conflicts of Interest

The authors declare no conflicts of interest.

## Supporting information


**Table S1:** Distribution of children's responses to the activities in the original CAPE.
**Table S2:** Item‐by‐item translation and adaptation process of the Danish CAPE/PAC.

## Data Availability

The data underlying this study are available from the corresponding author upon reasonable request, in accordance with ethical and legal requirements.
