# Supplementary material for: Cross‐Cultural Adaptation of Children's Assessment of Participation and Enjoyment (CAPE) and Preferences for Activities of Children (PAC) in a Danish Context
Source: Child Care Health Dev. 2026 Nov 6;52(6):e70377. doi: 10.1111/cch.70377 (PMC13743912; doi:10.1111/cch.70377)
Supplement: Supplementary file 1 [file CCH-52-e70377-s001.docx]

**Supplementary A – Distribution of children’s responses to the activities in the original CAPE**

**Table S1** Distribution of children’s responses to the activities in the original CAPE.

| **Activity type** | **Item** | **Activity** | **Engaging in activities***, n (%)* | | |
| --- | --- | --- | --- | --- | --- |
|  |  |  | TDC *n=107* | CWI *n=17* | In total  *n=124* |
| Recreational | 1 | Puzzle | 36 (34) | 6 (35) | 42 (34) |
|  | 2 | Playing cards | 99 (93) | 16 (94) | 115 (93) |
|  | 3 | Doing crafts | 80 (75) | 13 (77) | 93 (75) |
|  | 4 | Collecting things | 56 (52) | 8 (47) | 64 (52) |
|  | 5 | Playing on computer | 99 (93) | 15 (88) | 114 (93) |
|  | 12 | Playing with pets | 90 (84) | 13 (77) | 103 (83) |
|  | 14 | Doing pretended play | 39 (36) | 7 (41) | 46 (37) |
|  | 15 | Playing with toys | 49 (46) | 10 (59) | 59 (48) |
|  | 32 | Going for a walk | 95 (89) | 16 (94) | 111 (90) |
|  | 36 | Playing on equipment | 70 (65) | 10 (59) | 80 (65) |
|  | 44 | Watching TV/rented movie | 105 (98) | 15 (88) | 120 (97) |
|  | 55 | Taking care of a pet | 79 (74) | 11 (65) | 90 (73) |
| Physical | 16 | Doing martial arts | 26 (24) | 1 (6) | 27 (22) |
|  | 20 | Racing track or field | 29 (27) | 2 (12) | 31 (25) |
|  | 21 | Doing team sports | 79 (74) | 17 (100) | 96 (77) |
|  | 30 | School clubs | 51 (48) | 9 (53) | 60 (48) |
|  | 33 | Bicycling, skateboard, inlines | 89 (83) | 9 (53) | 98 (79) |
|  | 34 | Water sports | 22 (21) | 2 (12) | 24 (19) |
|  | 35 | Snow sports | 52 (49) | 2 (12) | 54 (44) |
|  | 37 | Playing games | 91 (85) | 16 (94) | 107 (86) |
|  | 38 | Gardening | 46 (43) | 7 (41) | 53 (43) |
|  | 40 | Doing individual physical activity | 75 (70) | 5 (29) | 80 (65) |
|  | 41 | Playing non-team sports | 39 (36) | 3 (18) | 42 (34) |
|  | 51 | Doing a paid job | 51 (48) | 2 (12) | 53 (43) |
| Social | 6 | Talking on the phone | 106 (99) | 15 (88) | 121 (98) |
|  | 7 | Going to a party | 86 (80) | 12 (71) | 98 (79) |
|  | 8 | Hanging out | 96 (90) | 11 (65) | 107 (86) |
|  | 9 | Visiting | 107 (100) | 15 (88) | 122 (98) |
|  | 11 | Entertaining others | 104 (97) | 16 (94) | 120 (97) |
|  | 42 | Going to a movie | 83 (78) | 13 (77) | 96 (77) |
|  | 45 | Going to a live event | 37 (35) | 5 (29) | 42 (34) |
|  | 46 | Going on a full-day outing | 63 (59) | 14 (82) | 77 (62) |
|  | 48 | Listening to music | 102 (95) | 15 (88) | 117 (95) |
|  | 52 | Making food | 87 (81) | 13 (77) | 100 (81) |

Abbreviations: typically developing children **(TDC)** and children with impairments **(CWI)**.

**Table S1** *(Continued)*

| **Activity type** | **Item** | **Activity** | **Engaging in activities***, n (%)* | | |
| --- | --- | --- | --- | --- | --- |
|  |  |  | TDC  *n=107* | CWI  *n=17* | In total *n=124* |
| Skill-based | 17 | Swimming | 58 (54) | 14 (82) | 72 (58) |
|  | 18 | Doing gymnastics | 47 (44) | 4 (24) | 51 (41) |
|  | 19 | Riding (a horse) | 6 (6) | 5 (29) | **11 (9)*** |
|  | 22 | Learning to sing | 14 (13) | 2 (12) | 16 (13) |
|  | 23 | Taking art lessons | 4 (4) | 2 (12) | **6 (5)*** |
|  | 24 | Learning to dance | 27 (25) | 1 (6) | 28 (23) |
|  | 26 | Playing an instrument | 31 (29) | 4 (24) | 35 (28) |
|  | 27 | Taking music lessons | 10 (9) | 0 (0) | **10 (8)*** |
|  | 28 | Community organizations | 20 (19) | 4 (24) | 24 (19) |
|  | 31 | Dancing | 66 (62) | 5 (30) | 71 (57) |
| Self-improvement | 10 | Writing letters | 38 (36) | 2 (12) | 40 (32) |
|  | 13 | Writing a story | 31 (29) | 4 (24) | 35 (28) |
|  | 25 | Getting extra help with schoolwork | 45 (42) | 2 (12) | 47 (38) |
|  | 29 | Doing religious activity | 25 (23) | 3 (18) | 28 (23) |
|  | 43 | Going to library | 30 (28) | 7 (41) | 37 (30) |
|  | 47 | Reading | 90 (84) | 11 (65) | 101 (82) |
|  | 49 | Doing volunteer work | 23 (22) | 2 (12) | 25 (20) |
|  | 50 | Doing a chore | 94 (88) | 14 (82) | 108 (87) |
|  | 53 | Doing homework | 100 (94) | 8 (47) | 108 (87) |
|  | 54 | Shopping | 90 (84) | 10 (59) | 100 (81) |

*****Indicates that less than 10% of the total cohort participated in the activity. Abbreviations: typically developing children **(TDC)** and children with impairments **(CWI)**.

**Supplementary B - Written report regarding the cross-cultural adaptation of the CAPE/PAC**

An advisory board is involved in all steps of the process and includes:

- A pediatric occupational therapist, an end-user (parent of a child with a disability)
- A pediatric physiotherapist, Mette Johansen
- Two professors in pediatrics (Christina Hoei-Hansen and Søren Hagstrøm)
- MD, PhD student Michelle Østergaard
- Pediatrician, PhD, pos.doc Ninna Brix (project manager)
- Medical doctor Ida Nørgaard

The translation process of the CAPE/PAC questionnaire from English to Danish consisted of the following steps:

First, written permission was obtained from CanChild to translate the Children’s Assessment of Participation and Enjoyment and the Preferences for Activities of Children (CAPE & PAC) materials into Danish. Three people from the advisory board (Michelle, Ninna, and Mette) translated the English version into Danish. As a result of a consensus meeting to address discrepancies, a synthesis of the three translations was prepared. The synthesized version was then translated back into English by two experienced bilingual translators (Jo Sturgess and Clea Mosetsanagape Petreman), both of whom have English as their native language.

The original CAPE/PAC was developed in Canada, and cultural differences between Canada and Denmark necessitated research into the relevance of the CAPE/PAC activity items within Danish culture. Therefore, semi-structured group interviews were conducted in a Danish public school across five grades: second, fourth, sixth, and ninth. One of the authors facilitated the interviews, while another took notes on the activities discussed. The students were asked about the activities they participated in outdoors and indoors outside school during spring/summer and autumn/winter. A similar semi-structured interview was conducted with eight parents of children and adolescents with disabilities playing in LykkeLiga; a training offer adapted to children and adolescents with disabilities. The interview took place in the cafeteria during the training. Following the training, some children and adolescents with disabilities were also asked about outdoor and indoor activities outside school during spring/summer and autumn/winter.

The interviews generated a long list of leisure activities, which was compared to the 55 original activity items in CAPE/PAC by two individuals. Any discrepancies were resolved by consensus, and 24 activities that did not align with the original activities were listed separately as possible new activities.

After the back translations and the cultural adaptation into a Danish context were made, a harmonization committee, consisting of the advisory board members and the backward translators, met to discuss and harmonize discrepancies. The following items were discussed:

The wording of the possible answer types was discussed to ensure sufficient variation in phrasing for the child to grade them, and cultural adaptations were made where necessary. “Somewhat; Sort of” was translated to “Lidt” as the most common Danish term for something in between “Definitely no” and “Definitely yes”. Additionally, “In your community” and “Beyond your community” were translated as “I dit lokalmiljø” and “Udenfor dit lokalmiljø” respectively. The term “Lokalmiljø” was considered the word most likely to be understood by children, as a direct translation to “Samfund” could be somewhat ambiguous in a Danish context.

The translation and cultural adaptation of individual items are detailed in **Table S2**, along with the type of change made. The discrepancies were mainly categorized into three groups: 1) Linguistic, 2) Societal, and 3) Cultural.

**Table S2** Item-by-item translation and adaptation process of the Danish CAPE/PAC

|  | **Original English** | **T12 version** | **Back translation** | | **Final Danish version** | **Change** |
| --- | --- | --- | --- | --- | --- | --- |
|  |  |  | BT1 | BT2 |  |  |
| **PAC** | Front page, introduction  Record Form | Forside, introduktion, spørgeskema | Front page, introduction, questionnaire | Front page, introduction, questionnaire | Forside, introduktion, spørgeskema |  |
|  | Child’s Name | Barnets navn | Child’s name | Child’s name | Barnets navn |  |
|  | Date | Dato | Date | Date | Dato |  |
|  | Did the child require assistance to complete the PAC? Yes, no | Fik barnet hjælp til at udfylde spørgeskemaet, ja nej | Did the child get help to fill out the questionnaire? yes / no | Did the child need help to complete PAC? Yes / No | Fik barnet hjælp til at udfylde PAC? Ja, nej |  |
|  | Name of the person who assisted the child in completing the PAC | Navnet på den person, der hjalp med at udfylde PAC | Name of the person who helped fill out PAC | The name of the person who helped with completing PAC | Navnet på den person, der hjalp med at udfylde PAC |  |
|  | Relationship to child | Forhold til barnet | Relationship to the child | Relationship to the child | Forhold til barnet |  |
| **PAC directions** | Review each of the activities shown in the PAC by looking at the picture and reading the description of the activity provided | Gennemgå hver af de aktiviteter, der er vist i PAC ved at se på billedet og læse beskrivelsen af aktiviteten. | Go through each of the activities that are shown in PAC by looking at the picture and reading the description of the activity. | Go through each of the activities shown in PAC by looking at the picture and reading the description of the activity. | Gennemgå hver af de aktiviteter, der er vist i PAC ved at se på billedet og læse beskrivelsen af aktiviteten. |  |
|  | Ask the child if he or she could anything in the whole world, would he or she like to do the activity in the picture. It is important that the child pretend that he or she can do anything in the world… the sky is the limit | Spørg barnet, om han eller hun ville have lyst til at udføre aktiviteten på billedet, hvis han eller hun kunne gøre alt i hele verden. Det er vigtigt at barnet forestiller sig at kunne gøre alt i hele verden. Der er ingen grænser. | Ask the child, if he or she would want to do the activity in the picture, if he or she could do anything in the world. It is important that the child imagines being able to do anything in the world. There are no limits. | Ask the child, if he or she could do anything in the whole world, would they do the activity in the picture. It is important that the child imagines that he or she can do anything in the world… there are no limitations. | Spørg barnet, om han eller hun ville have lyst til at udføre aktiviteten på billedet, hvis han eller hun kunne gøre alt i hele verden. Det er vigtigt at barnet forestiller sig at kunne gøre alt i hele verden… der er ingen grænser. |  |
|  | Always let the child answer first, before offering any assistance. Remind the child that there are no right or wrong answers to the questions. | Lad altid barnet svare først inden der tilbydes hjælp.  Mind barnet om, at der ikke er nogen rigtige eller forkerte svar på spørgsmålene. | Always let the child answer first before offering help. Remind the child that there are no right or wrong answers to the questions. | Always let the child answer first, before you offer help. Remind the child that there are no right or wrong answers to the questions. | Lad altid barnet svare først inden der tilbydes hjælp.  Mind barnet om, at der ikke er nogen rigtige eller forkerte svar på spørgsmålene. |  |
|  | Circle the score associated with the child’s response. | Sæt en cirkel om den score, der svarer til barnets svar. | Circle the score that corresponds to the child’s answer. | Circle score that corresponds to the childs answer. | Marker den score, der matcher barnets svar. | Linguistic |
|  | Emphasize that this is not about the activities the child currently does, but about the activities he or she would like to do. | Understreg, at dette ikke handler om de aktiviteter, barnet aktuelt gør, men om de aktiviteter, han eller hun gerne vil gøre. | Emphasise that this isn’t about the activities that the child actually does, but about the activities he or she would like to do. | Emphasize that this is not about the activities that the child actually does, but the activities he or she wants to do. | Understreg, at dette ikke handler om de aktiviteter, barnet aktuelt gør, men om de aktiviteter, han eller hun gerne vil gøre. |  |
| **PAC example** | Here is an example of a completes item to show the child | Her er et eksempel på et udfyldt spørgsmål som kan vises til barnet | Here is an example of a completed question that can be shown to the child. | Here is an example of a completed item* to show the child: | Her er et eksempel på et udfyldt spørgsmål som kan vises til barnet |  |
|  | If you could do anything in the who world, would you like to be… skydiving  I would not like to do at all  I would sort of like to do  I would really like to do | Hvis du kunne gøre hvad som helst i hele verdenen, ville du så gerne… springe i faldskærm  Det ville jeg slet ikke  Det ville jeg måske gerne  Det ville jeg rigtig gerne | If you could do anything in the whole world, would you like to… go skydiving? I wouldn’t like to do it at all I would do it a little bit I would really like to do it | If you could do anything in the whole world, would you like to … go parachuting?  I don’t want to do that  I want to do that a little bit.  I really want to do that. | Hvis du kunne gøre hvad som helst i hele verdenen, ville du så gerne… springe i faldskærm  Det ville jeg slet ikke  Det ville jeg lidt  Det ville jeg rigtig gerne | Linguistic |
|  | If you could do anything in the whole world, would you like to be… | Hvis du kunne gøre hvad som helst i hele verdenen, ville du så gerne… | If you could do anything in the whole world, would you like to… | If you could do anything in the whole world, would you like to … | Hvis du kunne gøre hvad som helst i hele verdenen, ville du så gerne… |  |
| **Items** |  |  |  |  |  |  |
| 1 | Doing puzzled? | Lave puslespil | Do a jigsaw puzzle? | Do a puzzle | Lave puslespil |  |
| 2 | Playing board or card games? | Spille bræt- eller kortspil? | Play a board or card game? | Play boardgames or cards? | Spille bræt- eller kortspil? |  |
| 3 | Doing crafts, drawing or coloring? | Lave kreative aktiviteter, tegne eller farvelægge? | Do creative activities, draw or colour? | …do something creative, draw or color | Lave kreative aktiviteter, tegne eller farvelægge? | Linguistic |
| 4 | Collecting things? | Samle på ting? | Collect things? | Collect things | Samle på ting? |  |
| 5 | Playing computer or video games? | Spille computer- eller videospil? | Playing computer games, telephone, tablet Playstation, X-box? | Play computer games, telephone, tablet, playstation, X-box | Spille computer, telefon, tablet, PlayStation, Xbox? | Societal, Cultural (examples added) |
| 6 | Talking on the phone | Tale i telefon? | Talk on the telephone? E.g. calls, facetime etc. | Talk on the phone (e.g. calls, Facetime etc.) | Tale i telefon? F.eks opkald, FaceTime mm.? | Societal |
| 7 | Going to a party? | Gå til fest? | Go to a party? | Go to a party | Gå til fest? |  |
| 8 | Hanging out? | Hænge ud? | Hang out? | Hang out | Hænge ud? |  |
| 9 | Visiting? | Tage på besøg? | Visit someone | Visit | Besøge nogen (f.eks. til aftensmad eller en soveaftale)? | Cultural (examples added) |
| 10 | Writing letters? | Skrive breve? | Write letters? | Write letters | Skrive breve? |  |
| 11 | Entertaining others? | Underholde andre? | Entertain others? | Entertain others | Have gæster på besøg | Linguistic |
| 12 | Playing with pets? | Lege med kæledyr? | Play with pets? | Play with pets | Lege med kæledyr? |  |
| 13 | Writing a story? | Skrive en historie? | Write a story? | Write a story | Skrive en historie? |  |
| 14 | Doing pretend or imaginary play? | Lege fantasi- eller rollespilsleg? | Play make-believe or role-playing games? | Play a fantasy or imaginary game | Lege fantasilege? | Linguistic |
| 15 | Playing with things or toys? | Lege med legetøj | Play with toys? | Play with things or toys | Lege med legetøj? | Linguistic |
| 16 | Doing martial arts? | Dyrke kampsport? | Do combat sports? | Do martial arts | Dyrke kampsport? |  |
| 17 | Swimming? | Svømme? | Swim? | Swim | Gå til svømning? | Linguistic |
| 18 | Doing gymnastics? | Lave gymnastik? | Do gymnastics? | Do gymnastics | Gå til gymnastik? | Linguistic |
| 19 | Horseback riding? | Ride på en hest? | Ride a horse? | Horseriding | Gå til ridning? | Linguistic |
| 20 | Racing or track and field? | Løbe eller deltage i atletik? | Run or do athletics? | Run or participate in track and field | Gå til løb eller atletik? | Linguistic |
| 21 | Doing team sports? | Dyrke holdsport? | Play team sports? | Join a team sport | Dyrke holdsport? |  |
| 22 | Learning to sing (choir or individual lessons)? | Lære at synge (kor eller individuelle timer)? | Learn to sing (choir or individual lessons)? | Learn to sing (choir or private lessons) | Lære at synge (kor eller individuelle timer)? |  |
| 23 | Taking art lessons? | Gå til billedkunst | Go to art lessons? | Take art lessons | Gå til billedkunst | Cultural  (removed) |
| New 23 |  |  |  | Doing creative activities online (e.g. programming and making music) | Lave kreative digitale aktiviteter (f.eks. programmere og musik) | Cultural (replaced with item 23) |
| 24 | Learning to dance? | Lære at danse? | Learn to dance? | Learn to dance | Gå til dans? | Linguistic |
| 25 | Getting extra help for schoolwork from a tutor? | Få ekstra hjælp til lektier fra en tutor? | Get extra help with schoolwork from a tutor? | Get extra help with school work from a tutor | Få ekstra hjælp til lektier af en tutor/vejleder? | Linguistic |
| 26 | Playing a musical instrument? | Spille på et instrument? | Play a musical instrument? | Play a musical instrument | Spille på et musikalsk instrument? |  |
| 27 | Taking music lessons? | Tage musiktimer? | Take music lessons? | Take music lessons | Få musik undervisning? | Cultural (removed) |
| New 27 |  |  |  | Going to a café/restaurant | Spist på en restaurant eller cafe | Cultural (replaced with item 27) |
| 28 | Participating in community organizations? | Deltage I lokalt foreningsarbejde? | Take part in local association work? | Participate in a community organization | Deltage i lokale foreninger (f.eks. spejder)? | Linguistic |
| 29 | Doing religious activity? | Deltage i religiøse aktiviteter? | Participate in religious activities? | Participate in a religious activity | Deltage i religiøse aktiviteter? |  |
| 30 | Participating in school clubs? | Deltage I skoleklubber? | Join school clubs? | Participate in a school club | Deltage i SFO/DUS og/eller andre fritidsklubber på skolen | Linguistic |
| 31 | Dancing | Danse? | Dance? | Dance | Danse? |  |
| 32 | Going for a walk or a hike? | Gå en tur eller vandre? | Go for a walk or go hiking? | Go for a walk or hike | Gå en tur eller vandre? |  |
| 33 | Bicycling, in-line skating, or skateboarding? | Cykle, stå på rulleskøjter eller skateboard? | Bike, inline skate or skateboard? | Bike, in-line skate or skateboarding | Cykle, stå på rulleskøjter eller skateboard? | Linguistic |
| 34 | Doing water sports? | Dyrke vandsport? | Do watersports? | Join a water sport | Dyrke vandsport (f.eks. svømme, surfe, vandski) | Cultural (examples added) |
| 35 | Doing snow sports? | Dyrke vintersport? | Do winter sports? | Join a winter sport | Dyrke vintersport (f.eks. ski, skøjte og kælke)? | Linguistic, cultural (examples added) |
| 36 | Playing on equitment? | Lege på legeplads? | Play on a playground? | Play on equipment | Lege på udstyr (f.eks.. trampolin, gynge, rutchebane)? | Linguistic |
| 37 | Playing games? | Spille spil? | Play a game? | Play games | Boldspil og leg (f.eks. basket, rundbold, fangeleg)? | Cultural (examples added) |
| 38 | Gardening? | Lave havearbejde? | Do gardening? | Garden work | Lave havearbejde? |  |
| 39 | Fishing? | Fiske? | Go fishing? | Fishing | Fiske? |  |
| 40 | Doing individual physical activities? | Lave fysiske aktiviteter alene | Do physical activities alone? | Participate in an individual physical activity | Lave fysiske aktiviteter alene (f.eks. løbe, yoga, styrketræne)? | Cultural (examples added) |
| 41 | Playing non-team sports? | Dyrke ikke-holdsport? | Play non-team sports? | Join a non-team sport | Dyrke sport der ikke er holdsport? |  |
| 42 | Going to the movies? | Gå i biografen? | Go to the cinema? | Go to the theater | Gå i biografen? |  |
| 43 | Going to the public library? | Gå på biblioteket | Go to the library? | Go to the public library | Gå på biblioteket | Linguistic |
| 44 | Watching TV or a rented movie? | Se tv eller film? | Watch TV or a film? | See tv or a film rental | Se tv eller film? | Societal |
| 45 | Going to a live event? | Tage til koncert? | Go to a concert? | Go to a live event | Tage til live beginhed (f.eks. koncert, teater eller sport)? | Cultural (examples added) |
| 46 | Going on a full-day outing? | Tage på en heldags udflugt? | Go on a day trip? | Go on a whole day outing | Tage på en heldags udflugt (f.eks. Zoo, stranden, picnic)? | Cultural (examples added) |
| 47 | Reading? | Læse? | Read? | Read | Læse? |  |
| 48 | Listening to music? | Lytte til musik? | Listen to music? | Listen to music | Lytte til musik? |  |
| 49 | Doing volunteer work? | Lave frivilligt arbejde? | Do charity work? | Do volunteering work | Lave frivilligt arbejde? |  |
| 50 | Doing chore? | Lave huslige pligter? | Do household chores? | Do chores | Hjælpe til derhjemme (f.eks. støvsuge, passe søskende)? | Cultural (examples added) |
| 51 | Doing a paid job? | Udføre et betalt arbejde? | Do paid work? | Have a job | Arbejde og få betaling | Linguistic |
| 52 | Making food? | Lave mad? | Cook? | Make food (cook) | Lave mad eller bage? | Cultural (examples added) |
| 53 | Doing homework? | Lave lektier? | Do homework? | Do homework | Lave lektier? |  |
| 54 | Shopping? | Shoppe? | Go shopping? | Shop | Shoppe? |  |
| 55 | Taking care of a pet? | Passe et kæledyr? | Care for a pet? | Take care of pets | Tage sig af et kæledyr? |  |
| **CAPE** | Front page, introduction, record form | Forside, introduktion, spørgeskema | Front page, introduction, questionnaire | Front page, introduction, questionnaire | Forside, introduktion, spørgeskema |  |
|  | Child’s name | Barnets navn | Child’s name | Child’s name | Barnets navn |  |
|  | Date | Dato | Date | Date | Dato |  |
|  | Did the child reuire assistance to complete the CAPE? Yes, no | Fik barnet hjælp til at udfylde spørgeskemaet?  ja nej | Did the child get help to fill out the CAPE? yes / no | Did the child require help to complete CAPE? Yes/No | Fik barnet hjælp til at udfylde CAPE?  ja nej |  |
|  | Name of the person who assisted the child in completing the CAPE | Angiv navnet på personen der hjalp barnet med at besvare CAPE | Enter the name of the person who helped the child answer CAPE | The name of the person who helped with completion of CAPE | Angiv navnet på personen der hjalp barnet med at besvare CAPE |  |
|  | Relationship to child | Relation til barnet | Relationship to the child | Relationship to the child | Forhold til barnet |  |

| **CAPE directions** | Review each of the activities shown in the CAPE by looking at the picture and reading the description of the activity provided | Gennemgå hver af de aktiviteter, der er vist i CAPE ved at se på billedet og læse beskrivelsen af aktiviteten. | Go through each of the activities that are shown in CAPE by looking at the picture and reading the description of the activity. | Go through each of the activities shown in CAPE by looking at the picture and reading the description of the activity. | Gennemgå hver af de aktiviteter, der er vist i CAPE ved at se på billedet og læse beskrivelsen af aktiviteten. |  |
| --- | --- | --- | --- | --- | --- | --- |
|  | For each activity, ask the child if he or she has done the activity in the past months. It may be helpful for the child to think of an activity or event that happened approximately four months ago (e.g birthday, holiday, spring break, start or end of school year, or a cultural or religious event celebrated by the child’s family) or to look at a calender. | Spørg barnet om han eller hun har lavet aktiviteten indenfor de sidste måneder. Det kan måske være en hjælp for barnet at tænke tilbage på en aktivitet eller begivenhed der skete for cirka fire måneder siden (fx en fødselsdag, rejse, sommerferie, starten eller slutningen af et skoleår eller en kulturel eller religiøs begivenhed i barnets familie) eller se på en kalender | Ask the child if he or she has done the activity within the past four months. It may be helpful to the child to think back to an activity or event that happened approximately four months ago (eg. a birthday, trip, summer holiday, start or end of a school year or a cultural or religious event in the child’s family) or to look at a calendar. | For each activity, ask the child if he or she has done the activity in the last four months. It can be useful for the child to think of an activity or event that happened roughly four months ago (e.g. birthday, holiday, spring vacation, beginning or end of the school year or a cultural or religious event celebrated in the child’s family) or to look at a calendar. | Spørg barnet om han eller hun har lavet aktiviteten indenfor de sidste måneder. Det kan måske være en hjælp for barnet at tænke tilbage på en aktivitet eller begivenhed der skete for cirka fire måneder siden (fx en fødselsdag, rejse, sommerferie, starten eller slutningen af et skoleår eller en kulturel eller religiøs begivenhed i barnets familie) eller se på en kalender |  |
|  | If the child has not done the activity in the last four months, check No and go to the next item. | Hvis barnet ikke har udført aktiviteten i de sidste fire måneder, marker "Nej" og gå til næste punkt. | If the child has not done the activity in the last four months, mark “No” and move on to the next item. | If the child has not done the activity during the last four months, check ”No” and go to the next item. | Hvis barnet ikke har udført aktiviteten i de sidste fire måneder, marker "Nej" og gå til næste punkt. |  |
|  | If the child has done the activity in the last four months, check Yes and complete the rest of the questions for that item. Circle the scores associated with the child’s response. | Hvis barnet har lavet aktiviteten inden for de sidste fire måneder, sæt et kryds i Ja og besvar resten af spørgsmålene for punktet. Marker de scores der passer til barnets svar. | If the child has done the activity within the last four months, check Yes and answer the rest of the questions for the item. Mark the scores that correspond to the child’s answer. | If the child has done the activity during the last four months, check ”Yes” and fill out the rest of the questions for that item. Circle the score that corresponds to the childs answer. | Hvis barnet har lavet aktiviteten inden for de sidste fire måneder, sæt et kryds i Ja og besvar resten af spørgsmålene for punktet. Sæt et kryds ved de scores der passer til barnets svar. |  |
|  | Always let the child answer first, before offering any assistance. Remind the child that there are no right or wrong answers to the questions. | Lad altid barnet svare først inden der tilbydes hjælp. Mind barnet om at der ikke er nogen rigtige eller forkerte svar | Always let the child answer first before offering help. Remind the child that there are no right or wrong answers. | Always let the child answer first, before you offer help. Remind the child that there are no right or wrong answers to the questions. | Lad altid barnet svare først inden der tilbydes hjælp. Mind barnet om at der ikke er nogen rigtige eller forkerte svar |  |
| **CAPE example** | Here is an example of a completed item to show the child | Her er et eksempel på et besvaret spørgsmål til at vise barnet | Here is an example of an answered question to show the child | Here is an example of a completed item to show the child: | Her er et eksempel på et besvaret spørgsmål til at vise barnet |  |
|  | Ask the child if he or she has brushed his or her teeth in the past four months | Spørg barnet om han eller hun har børstet sine tænder de sidste fire måneder | Ask the child if he or she has brushed their teeth in the last four months. | Ask the child if he or she has brushed their teeth in the last four months. | Spørg barnet om han eller hun har børstet sine tænder de sidste fire måneder |  |
|  | Brushing your teeth | Børstet dine tænder | Brushed your teeth | Toothbrushing | Børstet dine tænder |  |
|  | Have you done this activity in the past four months?  No (go to the next item)  Yes (complete each of the questions below) | Har du udført denne aktivitet inden for de seneste fire måneder?  Nej (gå til næste punkt)  Ja (besvar alle spørgsmålene nedenfor) | Have you done this activity within the last four months? No (go to the next item) Yes (answer all the questions below) | Have you done this activity in the last four months?  No (go to the next item)  Yes (complete each of the questions below) | Har du udført denne aktivitet inden for de seneste fire måneder?  Nej (gå til næste punkt)  Ja (besvar alle spørgsmålene nedenfor) |  |
|  | Ask the child how often he or she brushes her teeth | Spørg barnet hvor ofte han eller hun børster sine tænder | Ask the child how often he or she brushes his or her teeth | Ask the child, how often he or she brushes teeth: | Spørg barnet hvor ofte han eller hun børster sine tænder |  |
|  | How often?  1 time in the past 4 months  2 time in the past 4 months  1 time a month  2-3 times a month  1 time a week  2-3 times a week  1 time a day or more | Hvor ofte?  1 gang i de sidste 4 måneder  2 gange de sidste 4 måneder  1 gang om måneden  2-3 gange om måneden  1 gang om ugen  2-3 gange om ugen  1 gang om gangen eller mere | How often? Once in the last 4 months Twice in the last 4 months Once a month 2-3 times a month Once a week 2-3 times a week Once a day or more | How often?  once in the last four months.  twice in the last four months.  Once a month  2-3 times a month  Once a week  2-3 times a week  Once a day? Or more | Hvor ofte?  1 gang i de sidste 4 måneder  2 gange de sidste 4 måneder  1 gang om måneden  2-3 gange om måneden  1 gang om ugen  2-3 gange om ugen  1 gang om gangen eller mere |  |
|  | Next, ask the child with whom he or she does this activity.  Note. If the child does an activity with two types of people, circle the type with the highest score. For example, if he or she goes to a party with sibling and friends, circle 4, With friend. If he or she does an activity with three or more types of people, for example, does an activity with family, other relatives, and friends, circle the score of 5, With others. | Spørg dernæst barnet hvem han eller hun laver denne aktivitet med. OBS hvis barnet udfører denne aktivitet med to forskellige personer, marker den person barnet laver det oftest med. F.eks hvis han eller hun går til et selskab med søskende og venner markeres 4: ”Med venner”. Hvis han eller hun laver aktiviteten med tre eller flere personer f.eks. laver en aktivitet med familie, andre slægtninge og venner marker scoren 5: ”Med andre” | Next, ask the child who he or she does the activity with. NOTE if the child does the activity with two different people, check the person the child does it with most often. Eg. if he or she goes to a party with siblings and friends, check 4: “With friends”. If he or she does the activity with three or more people, eg. does an activity with family, other relatives and friends, check the score 5: “With others” | Next, ask the child, who he or she does this activity with:  Note. If the child does an activity with two types of people, circle the type with the highest score.  For example, if he or she goes to a party with siblings and friends, circle 4, with friends. If he or she does an activity with 3 or more types of people, for example, does an activity with family, other relatives and friends, circle score 5, with others. | Spørg derefter barnet hvem han eller hun laver denne aktivitet med. Bemærk hvis barnet udfører denne aktivitet med to typer af personer, marker da den med den højeste score. F.eks. hvis han eller hun går til et fest med søskende og venner markeres 4: ”Med venner”. Hvis han eller hun laver aktiviteten med tre eller flere typer af personer, f.eks. laver en aktivitet med familie, andre slægtninge og venner marker scoren 5: ”Med andre” |  |
|  | With whom do you do this most often?  Alone  With family (Parents, brothers, Sisters)  With other Relatives (grandparents, Aunts, Uncles, Cousins)  With Friends  With Others (Instructors, Other individuals, or Multiple types of people) | Hvem laver du oftest denne aktivitet med  Alene  Med familie (forældre, brødre, søstre)  Med andre slægtninge (bedsteforældre, tanter, onkler, kusiner, fætre)  Med venner  Med andre (instruktører, andre personer eller forskellige grupper af personer | Who do you do this activity with most often? Alone With family (parents, brothers, sisters) With other relatives (grandparents, aunts, uncles, cousins) With friends With others (instructors, other people and different groups of people) | (With?) whom do you most often do this activity with?  alone  with family (parents, brothers, sisters)  with other relatives (grandparents, aunts, uncles, cousins).  with friends  with others (teachers, other individuals or other types of people) | Hvem laver du oftest denne aktivitet med  Alene  Med familie (forældre, brødre, søstre)  Med andre slægtninge (bedsteforældre, tanter, onkler, kusiner, fætre)  Med venner  Med andre (instruktører, andre personer eller forskellige typer af personer) |  |
|  | Next, ask the child where he or she does this activity  Note. If the child does the activity at school, it must be outside of regular classes, such as before or after school, at recess, or as part of the school club.  Neighborhood and Community may need to be defines for the child based on the type of community (urban og rural) in which he or she lives | Spørg derefter barnet, hvor aktiviteten bliver udført. OBS hvis barnet udfører aktiviteten i skolen, så skal det være udenfor den almindelige skoletid såsom før eller efter skole, i pauserne eller som en del af fritidstilbud.  Det kan være nødvendigt at definere nabolag og nærmiljø alt efter hvor barnet bor (storby eller landsby) | Next ask the child where the activity is carried out. NOTE if the child does the activity at school, it should be outside normal school hours, such as before or after school, during breaks or as part of an extracurricular activity. It may be necessary to define the neighbourhood or local environment, depending on where the child lives (city or village) | Next, ask the child, where he or she does this activity:  Note. If the child does the activity in school, it should be outside of normal school hours, as in before or after school, at breaktime or as part of a school club.  Neighborhood and community may need to be defined for the child based on the type of community (city or country) where he or she lives. | Spørg derefter barnet, hvor aktiviteten bliver udført. Bemærk hvis barnet udfører aktiviteten i skolen, så skal det være udenfor den almindelige skoletid, såsom før eller efter skole, i pauserne eller som en del af fritidstilbud.  Det kan være nødvendigt at definere nabolag og nærmiljø alt efter hvor barnet bor (storby eller landsby) |  |
|  | Where do you do this most often?  At Home  At a relative’s Home  In Your Neighborhood  At School (but not during classes)  In Your Community  Beyond Your Community | Hvor laver du oftest dette  Derhjemme  Ved familie  I dit nabolag  I skolen (men ikke i skoletid)  I dit nærmiljø  Udenfor dit nærmiljø | Where do you do this activity most often? At home At family’s In your neighbourhood At school (but not during school hours) In your local environment Outside your local environment | Where do you do this activity most?  Home  At a relatives house  In my neighborhood  At school  In my community  Outside of my community | Hvor laver du oftest aktiviteten  Derhjemme  Ved familie  I dit nabolag  I skolen (men ikke i skoletid)  I dit lokalmiljø  Udenfor dit lokalmiljø | Linguistic |
|  | Ask the child how much he or she likes doing the activity  Not at all  Somewhat; Sort of  Pretty much  Very much  Love it | Spørg barnet hvor meget han eller hun kan lide at lave aktiviteten  Slet ikke  Lidt  Nogenlunde  Rigtig meget  Elsker det | Next ask the child how much he or she likes to do the activity Not at all  A little  Fairly well Very much  Loves it | As the child, how much he or she likes? To do the activity:  Not at all  Somewhat; a bit  Quite a bit  Alot  Love it | Spørg barnet hvor meget han eller hun kan lide at lave aktiviteten  Slet ikke  Lidt  Meget  Rigtig meget  Elsker det |  |
| **Item 1-5** | Hobbies, Crafts, and Games  Items 1-5  These drawings show kids doing hobbies, crafts, or games. Think about any hobbies, crafts, or games that you have done in the past four months outside of school | Hobbyer, håndværk og spil:  spørgsmål 1-5  Disse billeder viser børn, der laver hobbyer, håndværk eller spiller spil. Tænk på en hobby, et håndværk eller spil du har spillet uden for skolen i de sidste fire måneder | Hobbies, crafts and games: questions 1-5 These pictures show children doing hobbies, crafts or playing games. Think of a hobby, craft or game you have played outside school in the last four months. | Hobbies, arts and crafts and games.  Items 1-5  These pictures show children who are doing hobbies, arts and crafts or games.  Think of possible hobbies, arts and crafts or games that you have done in the last four months outside of school. | Hobbyer, håndværk og spil:  Spørgsmål 1-5  Disse billeder viser børn, der laver hobbyer, håndværk eller spiller spil. Tænk på en hobby, et håndværk eller et spil du har spillet udenfor skolen de sidste fire måneder |  |
| **Item 6-11** | Social Activities  Items 6-11  The next drawings show kids doing things with other people. Think about the activities that you have done with other people in the past four months outside of school | Sociale aktiviteter  Spørgsmål 6-11  De næste tegninger forestiller børn der laver noget med andre mennesker. Tænk på aktiviteter som du har lavet med andre mennesker indenfor de sidste 4 måneder udenfor skoletid | Social activities Questions 6-11 The next drawings show children doing something with other people. Think of activities you have done with other people within the last 4 months outside school hours. | Social activities  Items 6-11  The next pictures show children who are doing things together with other people. Think of the activities that you have done together with other people in the last four months outside of school. | Sociale aktiviteter:  Spørgsmål 6-11  De næste billeder viser børn, der laver noget med andre mennesker. Tænk på aktiviteter, som du har lavet med andre mennesker udenfor skolen de sidste fire måneder |  |
| **Item 12-15** | Quiet Recreation  Items 12-15  These drawings show kids doing quiet activities that you have done in the past four months outside of school. | Rolige aktiviteter  Spørgsmål 12-15  Disse tegninger viser børn der laver rolige aktiviteter. Tænk på rolige aktiviteter som du har lavet med andre mennesker indenfor de sidste 4 måneder udenfor skoletid | Calm activities Questions 12-15 These drawings show children doing calm activities. Think of calm activities you have done the last 4 months outside school hours. | Quiet free time  Items 12-15  These pictures show children who are doing quiet activities. Think of the activities that you have done in the last four months outside of school. | Rolige aktiviteter:  Spørgsmål 12-15  Disse billeder viser børn, der laver rolige aktiviteter. Tænk på rolige aktiviteter, som du har lavet med andre mennesker udenfor skolen de sidste fire måneder |  |
| **Item 16-21** | Organized Sports  Items 16-21  In the next group of drawings, you will see kids playing different kinds of sports. The kids playing these sports are usually on sports teams and may play games against other teams. Sometimes these kids will have lessons or practices to get better at their sport. Think about any sports lessons, practices, or games that you have done in the past four months outside of school | Organiseret sport  Spørgsmål 16-21  I den næste gruppe af tegninger vil du se børn som deltager i forskellige sportsgrene. De børn der deltager i disse sportsgrene, er som regel på sportshold og vil måske spille kampe mod hinanden. Nogle gange får de børn undervisning eller øver sig i at blive bedre til sin sport.  Tænk på undervisning, træninger eller kampe, som du har deltaget i de sidste 4 måneder udenfor skoletid | Organised sport Questions 16-21 In the next group of drawings you will see children taking part in different sports. The children who take part in these sports are usually on a sports team and may want to play matches against each other. Sometimes these children have lessons or practice to get better at their sport. Think about lessons, practice or matches, that you have taken part in during the last 4 months outside school hours. | Organized sports  Items 16-21  In the next group of pictures, you will see children playing different types of sports. Children that play these sports are normally on a sports team and might play games against other teams. Sometimes, these children will have training or practice to get better at their sport.  Think of practice, training or games you have participated in, in the last four months outside of school. | Organiseret sport:  Spørgsmål 16-21  På de næste billeder vil du se børn, som deltager i forskellige sportsgrene. De børn der deltager i disse sportsgrene, er som regel på hold og vil måske spille kampe mod hinanden. Nogle gange får børnene undervisning eller øver sig i at blive bedre til sin sport.  Tænk på undervisning, træning eller kampe, som du har deltaget i udenfor skolen de sidste fire måneder |  |
| **Item 22-27** | Other Skill-Based Activities  Items 22-27  The next group of drawings shows kids taking lessons with an instructor or a tutor. The kids in these drawings are trying to get better at a skill or learn a new skill. These activities also include practices or rehearsals as well as performances such as recitals. Think about any lessons, practices, or performances that you have done in the past four months outside of school | Andre færdighedsbaserede aktiviteter  Spørgsmål 22-27  Den næste gruppe af tegninger viser børn, der får undervisning af en instruktør eller en underviser for at blive bedre til eller lærer en ny færdighed. Disse aktiviteter inkluderer både at øve sig og optrædener såsom koncerter. Tænk på alle lektioner, øvetimer eller optrædener du har haft de sidste fire måneder udenfor skolen. | Other skill-based activities Questions 22-27 The next group of drawings show children having lessons from an instructor or teacher to get better at or learn a new skill. These activities include both practicing and performances such as concerts. Think of all the lessons, practices or performances you have had the last four months outside of school. | Other skill based activities  Items 22-27  The next group of pictures shows children who are being taught by an instructor or a teacher, in order to get better at or learn a new skill. These activities include both practice and performances such as a concert. Think of all the lessons, practices or performances you have had in the last four months outside of school. | Andre færdighedsbaserede aktiviteter:  Spørgsmål 22-27  De næste billeder viser børn, der får undervisning af en instruktør eller en underviser for at blive bedre til eller lærer en ny færdighed. Disse aktiviteter inkluderer både at øve sig og optræde ved fx koncerter. Tænk på alle lektioner, træning eller optrædener, du har haft udenfor skolen de sidste fire måneder. |  |
| **Item 28-30** | Clubs, Groups, and Organizations  Items 28-30  These kinds of activities include any other club or group you might be involved in other than sports club. A club has to have a leader of some sort, and specific times that it meets. Think about any club or groups that you have been a part of in the past four months outside of school. | Fritidsklubber, grupper og foreninger  Spørgsmål 28-30  Disse aktiviteter inkluderer alle andre klubber eller grupper du kunne være en del af ud over sportsklubber. En klub skal have en form for leder og specifikke tidspunkter at mødes. Tænk på hvilken som helst klub eller gruppe du har været en del af i løbet af de sidste fire måneder udenfor skoletid | Leisure clubs, groups and associations Questions 28-30 These activities include all other clubs or groups you could be a part of besides sports clubs. A club must have some form of leader and specific meeting times. Think about any club or group you have been part of during the last four months outside school hours. | Clubs, groups and societies  Items 28 – 30  These activities include all other clubs or groups you could be a part of, other than sports clubs. A club should have some sort of leader and specific meeting times. Think of any club or group you have been a part of during the last four months, outside of schooltime. | Fritidsklubber, grupper og foreninger:  Spørgsmål 28-30  Disse aktiviteter inkluderer alle andre klubber eller grupper, du kunne være en del af ud over sportsklubber. En klub skal have en form for leder og specifikke tidspunkter at mødes. Tænk på hvilken som helst klub eller gruppe, du har været en del af udenfor skolen de sidste fire måneder. |  |
| **Item 31-41** | Active Physical Recreation  Items 31-41  These drawings show kids doing physical recreation activities. Some of the activities are sports that you would play, but these are not organized or team sports. Think about the physical recreation activities that you have done in the past four months outside of school | Aktive fysiske fritidsinteresser.  Spørgsmål 31-41  Disse tegninger viser børn som laver fysiske fritidsinteresser. Nogle af disse aktiviteter er sportsgrene, som du udfører, men de er ikke organiserede eller holdsport. Tænk på hvilke fysiske fritidsaktiviteter, som du har deltaget i indenfor de sidste fire måneder udenfor skoletid. | Active physical hobbies. Questions 31-41 These drawings show children doing physical hobbies. Some of these activities are sports, that you do, but they are not organized or team sports. Think about which physical hobbies you have taken part in within the last four months outside school hours. | Other physical free time activities  Items 31 – 41  These pictures show children who are doing physical activities in their free time. Some of these activities are types of sports you do, but they are not organized or team sports. Think of which physical activities you have participated in during the last four months, outside of school time. | Fysiske fritidsinteresser:  Spørgsmål 31-41  Disse billeder viser børn, som laver fysiske fritidsinteresser. Nogle af disse aktiviteter er sportsgrene, der ikke er organiserede eller holdsport. Tænk på hvilke fysiske fritidsaktiviteter, som du har deltaget i udenfor skolen de sidste fire måneder. |  |
| **Item 42-48** | Entertainment and Education  Items 42-48  The next group of drawings shows kids who are being entertained or learning new things. These activities do not include field trips that are taken with school. Think about activities you have done in the past four months where you were entertained or you learned something new outside school. | Underholdning og Uddannelse  Spørgsmål 42-48  Den næste gruppe tegninger viser børn der bliver underholdt eller lærer nye ting. Disse aktiviteter indeholder ikke udflugter som laves med skolen. Tænk på aktiviteter du har lavet indenfor de sidste fire måneder, hvor du blev underholdt eller lærte noget nyt udenfor skoletid | Entertainment and Education Questions 42-48 The next group of drawings show children being entertained or learning new things. These activities do not include school trips. Think about activities you have done within the last four months, where you were entertained or learned something new outside school hours. | Entertainment and education  Items 42- 48  The next group of pictures shows children who are being entertained or learning new things. These activities do not include school trips. Think of activities you have done during the last four months, where you were entertained or learned something new outside of school time. | Underholdning og uddannelse:  Spørgsmål 42-48  De næste billeder viser børn, der bliver underholdt eller lærer nye ting. Disse aktiviteter indeholder ikke udflugter, som laves med skolen. Tænk på aktiviteter du har lavet de sidste fire måneder, hvor du blev underholdt eller lærte noget nyt udenfor skoletid. |  |
| **Item 49-55** | Jobs, Chores, and Employment  Items 49-55  These activities are things kids do, like helping out at home. Think about any jobs or chores that you have done in the past four months outside of school. | Fritidsarbejde, pligter og ansættelser  Spørgsmål 49-55  Disse aktiviteter er ting som børn gør, så som at hjælpe til derhjemme.  Tænk på enhver form for fritidsarbejde eller pligter, som du har lavet inden for de sidste fire måneder udenfor skoletid | Part-time job, chores and employment Questions 49-55 These activities are things that children do, such as helping out at home. Think of any kind of part-time work or chores that you have done within the last four months outside school hours. | After school jobs, chores and employment  Items 49-55  These activities are things which children do, like helping out at home.  Think of any kind of job or chore which you have done during the last four months, outside of school time. | Fritidsarbejde, pligter og ansættelser:  Spørgsmål 49-55  Disse aktiviteter er ting som børn gør, f.eks. at hjælpe til derhjemme.  Tænk på enhver form for fritidsarbejde eller pligter, som du har lavet udenfor skoletid de sidste fire måneder. |  |
|  | Other Activities  Are there any other activities outside school that we have not talked about? This is an opportunity to add in any other activities you have done in the last four months that we have not asked you about. | Andre aktiviteter  Er der andre aktiviteter udenfor skolen, som ikke er blevet nævnt? Dette er muligheden for at tilføje aktiviteter, du har lavet de sidste fire måneder, som vi ikke har spurgt ind til. | Other Activities Are there any other activities outside of school that have not been mentioned? This is your chance to add any activities you have done in the past four months that we have not asked about. | Other Activities Are there any other out-of-school activities that have not been mentioned? This is your opportunity to include any activities you have done in the past four months that we have not asked about. | Andre aktiviteter  Er der andre aktiviteter udenfor skolen, som ikke er blevet nævnt? Dette er muligheden for at tilføje aktiviteter, du har lavet de sidste fire måneder, som vi ikke har spurgt ind til. |  |

**1. TRANSLATION-RELATED ADAPTATIONS**

The rationale for the changes in the translation process is described in detail below.

**1.1 Linguistic**

The direct translation of some questions may not be easily understood by Danish children due to differences in phrasing and wording. To address this, additional written examples were incorporated to clarify the questions or activities, and some questions were rephrased to align with Danish language conventions while preserving their original meaning. This applies to the following items:

Item 3: “Doing crafts, drawing or coloring” was translated to “Lave kreative aktiviteter, tegne eller farvelægge”.

Item 11: “Entertaining others” was translated to “Have gæster på besøg” as it includes hosting friends and family for a meal, party, or gathering.

Item 14: “Doing pretend or imaginary play” was translated to “Lege fantasilege”.

Item 15: “Playing with things and toys” was translated to “Lege med legetøj”.

Item 20: “Racing or track and field” was translated to “Gå til atletik eller løb” to ensure children think of racing.

Item 25: “Getting extra help for schoolwork from a tutor” was translated to “Få ekstra hjælp til lektier fra en tutor/vejleder” as we do not expect all children to know what a tutor is. “Vejleder” is a more common Danish word to describe a person who guides in an educational setting.

Item 33: “Bicycling, in-line-skating or skateboarding” was translated to “Cykle, stå på rulleskøjter eller skateboard”.

Item 35: “Doing snow sports” was translated to “Dyrke vintersport (f.eks. skiferie, skøjte og kælke)”.

Item 36: “Playing on equipment” was translated to “Lege på udstyr (f.eks. trampolin, gynge, rutchebane)”.

Item 43: “Going to the public library” was translated to “Gå på biblioteket”.

Item 51: “Having a paid job” was translated to “Arbejde og få betaling”.

During the harmonization process, consensus was reached that certain cultural adjustments were necessary to ensure the intended meaning of the questions remained intact. The following activity items were revised:

Item 17: “Swimming” was translated to “Gå til svømning” to ensure children think of an organized sport.

Item 18: “Doing gymnastics” was translated to “Gå til gymnastik” to ensure children think of an organized sport.

Item 19: “Horseback riding” was translated to “Gå til ridning” as horseback riding in Denmark typically takes place within an organized setting.

Item 24: “Learning to dance” was translated to “Gå til dans” as this translation refers to learning to dance with help from an instructor.

Item 28: “Participating in community organizations” was translated to “Deltage i lokale foreninger (fx spejder)” as community organizations in Denmark, apart from scouting, are quite limited.

Item 30: “Participating in school clubs” was translated to “Deltage I SFO/DUS og/eller andre fritidsklubber på skolen” as the concept of school clubs differs in Denmark.

**1.2 Changing times**

The original CAPE/PAC questionnaire was developed in 2004, and some questions have become outdated, particularly those related to technology. In these cases, contemporary examples were incorporated to ensure relevance while preserving the original concepts and meanings of the items or questions. In **Table S2**, “societal change” refers to adaptations made to reflect changing times. This applies to the following items:

Item 5: “Playing computer or video games” was translated to “Spille på computer, telefon, tablet, PlayStation, X-box” as most children in Denmark today do not know the word “videogame”.

Item 6: “Talking on the phone” was translated to “Tale i telefon (f.eks. opkald, FaceTime mm.)”.

Item 44: “Watching TV or a rented movie” was translated to “Se TV eller film”.

**2. CULTURAL ADAPTATION**

To determine which items to include in the proposed Danish version of CAPE/PAC, we decided to include only activities performed by more than 10% of participants. This led to three items from the original version being excluded:

Item 23: “Taking art lessons” (5%)

Item 27: “Taking music lessons” (8%)

Item 19: “Horseback riding” (9%). Although only 9% of the total cohort did this activity, we decided to keep it. In LykkeLiga, 5/17 (29%) of children reported doing the activity.

**2.1 New activities**

Of the 24 new activities, four met the criteria for inclusion in the Danish version. However, to keep the total number of activities at 55, we added only two, as the remaining two could be included as examples in the original activities.

Item 23 was replaced with ”Lave kreative digitale aktiviteter (f.eks. programmere og musik)” / Doing creative activities online (e.g. programming and making music).

Item 27 was replaced with “Spist på en restaurant eller cafe/”Going to a café/restaurant”.

**2.2 Examples added**

Subsequently, it was decided that 12 of the 24 new activities that also met the criteria fit into the original activities. Therefore, it was decided to include some of them as examples under the original activities. This applies to the following:

Item 9: “Visiting”. Examples were added because 68% of Danish children responded “yes” to “Haft en soveaftale”/”Had a sleepover”. This led to the final translation “Besøge nogen (f.eks. til aftensmad eller en soveaftale)?”/”Visiting (e.g. for dinner or a sleepover)”.

Item 34: “Doing water sports”: examples were added because 80% of Danish children responded “yes” to ”Badet eller svømmet i svømmehal, havet, swimmingpool eller spabad”/”Bathed or swum in a swimming hall, the sea, a swimming pool, or a hot tub”. This led to the final translation: “Dyrke vandsport (f.eks. svømme, surfe, vandski)”/”Doing water sports (e.g. swimming, surfing, water skiing)”.

Item 37: “Playing games”: examples were added because 28% of Danish children responded “yes” to “Rundbold”/”Rounders” and 59% responded “yes” to “Fangeleg”/”Tag”. This led to the final translation: “Boldspil og leg (f.eks. basket, rundbold, fangeleg)”/”Playing games (e.g. basket, rounders, tag)”.

Item 40 “Doing individual physical activities”: examples were added because 58% of Danish children responded “yes” to “Løbetrænet”/”Running (alone)”. This led to the final translation: “Lave fysiske aktiviteter alene (f.eks. yoga, løbet og styrketræne)”/”Doing individual physical activities (e.g. yoga, running, strength training) ”.

Item 45: “Going to a live-event”: examples were added because 63% of Danish children responded “yes” to “Været ude og se en håndboldkamp/fodboldkamp/ishockeykamp”/”Attended a handball, football or ice hockey match”. This led to the final translation: “Tage til live begivenhed (f.eks. koncert, teater, sport)”/”Going to a live-event (e.g. concert, theater, sports)”.

Item 46: “Going on a full-day outing”: examples were added because 21% of Danish children responded “yes” to “Været på picnic”/”Going to a picnic”, 61% “Været på stranden” and 75% “været I skoven”. This led to the final translation: ”Tage på en heldagsudflugt (f.eks. zoo, stranden, picnic)”/”Going on a full-day outing (e.g. zoo, beach, picnic)”.

Item 50: “Doing chores”: examples were added because 45% of Danish children responded “yes” to “Passet søskende”/”Taking care of a sibling”. This led to the final translation: “Hjælpe til derhjemme (f.eks. støvsuge, passe søskende)”/”Doing chores (e.g. vacuuming, taking care of siblings)”.

Item 52: ”Making food”: examples were added because 76% of Danish children responded “yes” to “Har bagt”/”Been cooking”. This led to the final translation: ”Making food or cooking”.

“Bruge telefon eller iPad”/”Use telephone or iPad” was included as an example in item 5

**2.3 Not included activities**

10 of the new activities technically met the criteria for inclusion in the Danish version, however, the research group decided not to include them for various reasons:

“Kørt på motorcross”/”Rode on a motocross bike”, “Kørt på scooter”/”Rode on a scooter”, and ”Kørt på elløbehjul”/”Rode on an electric scooter” were considered irrelevant, as they are not considered physical activities, nor do they contribute to socializing.

“Snakket med familie derhjemme”/”Talked with family at home”, “Hygget med slik/vafler/is eller andet”/”Enjoyed sweets/waffles/ice cream or something else”, and ”Hygget med kakao”/”Enjoyed warm cocoa” were considered irrelevant, as they do not provide any insight into children’s participation in activities.

“Været i sommerhus”/”Stayed in a summer house”, “Været på sommerferie”/”Went on summer vacation”, and “Været på skiferie” were considered irrelevant, as holidays do not provide specific information about children’s participation in activities.

“Været på lejrtur/koloni”/”Went on a camp trip/colony” was considered irrelevant, as camp trips/colonies are often organized by schools in Denmark.
